# Distribution of Iron on FCC Catalyst and Its Effect on Catalyst Performance

**DOI:** 10.3389/fchem.2021.640413

**Published:** 2021-03-08

**Authors:** Yitao Liao, Tao Liu, Xiaohui Du, Xionghou Gao

**Affiliations:** ^1^ College of Chemistry and Chemical Engineering, Northwest Normal University, Lanzhou, China; ^2^ Lanzhou Petrochemical Research Center, Petrochemical Research Institute, PetroChina, Lanzhou, China

**Keywords:** fluid catalytic cracking, iron contamination, nodulation, cyclic deactivation, iron naphthenate

## Abstract

The effects of different iron contamination content on the formation of iron nodules and the performance of FCC catalysts have been studied by cyclic deactivation treatment using iron naphthenate. The catalysts were characterized by X-ray diffraction, N2 adsorption-desorption, and SEM. The catalysts’ performance was evaluated by the Advanced Cracking Evaluation device. It has been found that there will be obvious nodulation on the catalyst when the iron concentration exceeds 7,400 μg/g. With the iron deposition from 53 μg/g to 11,690 μg/g, the crystal structure of zeolite will not be destroyed by iron. The surface area and pore volume of the catalyst decreased significantly; the surface area decreased from 125.3 m^2^/g to 91.0 m^2^/g, and the pore volume decreased from 0.21 cm^3^/g to 0.16 cm^3^/g. The studies also showed that the increase of iron deposition will lead to the decrease of catalytic reaction efficiency. With the iron deposition from 53 μg/g to 11,690 μg/g, the conversion decreased by 4.83%. Under the same 78 wt.% conversion, bottoms yield and coke yield increased by 2.15% and 1.31%, while gasoline yield and LCO yield decreased by 2.59% and 2.16%, respectively. The real state of the industrial iron contaminated equilibrium catalyst can be mimicked by using the cyclic deactivation method.

## Introduction

Fluid catalytic cracking (FCC) technology is one of the most important conversion processes for efficient residue processing, and it plays an important role in the oil refining field (Harding et al., 2001; Mehla et al., 2019). The FCC catalyst is widely used in the oil refining industry, and its performance affects the unit operation and the economic benefits of the refinery. Nickel, vanadium, iron, calcium, sodium, copper, and other metals exist in heavy crude oil in the form of porphyrin complexes, naphthenates, or inorganic compounds, which have toxic effects on the catalysts. With the increasing trend of heavy and poor quality of FCC feed stock, the influence of heavy metal deposition especially iron on the catalyst performance has gradually increased (Escobar et al., 2008; Liu et al., 2015; Souza et al., 2019). One common indicator of iron contamination is the advent of a particular type of particle morphology change, often referred to as “nodulation” (Rainer et al., 2003). According to the analysis of FCC unit operation, the increase of iron loading will lead to the decrease of apparent bulk density, the increase of slurry yield, and the deterioration of product distribution (Bai et al., 2018; Brandt et al., 2019; Zhou et al., 2020). Therefore, the study of iron deposition and its effect on catalytic cracking reaction is very important for continuous improvement of the FCC catalyst and guidance of industrial production.

In order to simulate the distribution of iron on the FCC catalyst and its effect on catalyst performance, it has been reported that iron deposition can be realized by the impregnating catalyst with iron compounds solution or by means of an improved fixed bed micro reactivity test (MAT) device (Lappas et al., 2001; Yaluris et al., 2004; Mathieu et al., 2014; Zhu et al., 2007). However, it is difficult to accurately simulate the state of the industrial iron contaminated equilibrium catalyst, which makes larger deviations from the experiment results to actual data.

The multi cyclic deactivation (MCD) unit can realize the deposition of heavy metals on the catalyst surface through multiple reaction and regeneration cycles so that the heavy metals deposited catalyst obtained in the laboratory can simulate the state of industrial equilibrium catalyst. The study of nickel and vanadium contamination by cyclic deactivation method has been reported elsewhere (Lappas et al., 2001; Long et al., 2005; Tangstad et al., 2008; Etim et al., 2016; Souza et al., 2018), but the report on the effect of iron contamination on the catalyst is less. The formation of iron nodules on the catalyst surface has not been successfully simulated in the existing reports, so it is difficult to truly reflect the actual effect of iron contamination (Rainer et al., 2003; Rainer et al., 2004; Jiang et al., 2018).

In this paper, iron deposition on the catalyst was realized by using the MCD unit. Effect of different amounts of iron deposition on the formation of iron nodules and physicochemical properties of FCC catalysts were investigated, which provided theoretical basis and experimental reference for further study of iron contamination reaction mechanism.

## Experimental

### Laboratory Deactivation

In this research, a commercial equilibrium catalyst (CAT-BASE see Table 1) was used to simulate iron contamination. In order to simulate the actual state of the iron contaminated equilibrium catalyst in FCC unit, different amounts of iron deposition and appropriate conditions have been selected to carry out cyclic deactivation method. Iron naphthenate (6% Fe) produced by Strem Chemicals Company was used as iron precursors.

**TABLE 1:** Characteristics of CAT-BASE.

	ω(Al_2_O_3_)%	ω(RE_2_O_3_)%	Ni (μg/g)	V (μg/g)	Fe (μg/g)
CAT-BASE	47.7	4.3	4,042	7,400	1,828

### Cyclic Deactivation

The catalyst was deactivated in MCDU, described in detail elsewhere (Tangstad et al., 2000; Luo et al., 2007; Psarras et al., 2007; Almas et al., 2019). The vacuum gas oil (VGO) spiked with iron naphthenate was prepared according to the target iron deposition amount. Then, 200 g catalyst was added to the quartz reactor to carry out iron cyclic deposition. The deactivated catalyst was treated through 200 cycles of cracking, stripping, regeneration, and cooling processes in the MCD unit. The cracking step was performed at a reaction temperature of 530°C and the regeneration step at 780°C. According to the different amounts of iron naphthenate spiked in the feed, the deactivated catalysts were labeled as LE-0 (Fe added in feed = 0 μg/g), LE-1 (Fe added in feed = 376 μg/g), LE-2 (Fe added in feed = 564 μg/g), LE-3 (Fe added in feed = 752 μg/g), and LE-4 (Fe added in feed = 1,128 μg/g).

### Catalyst Characterization

The metal content was determined by ZSX PrimusⅡX-ray fluorescence (XRF) instrument produced by Japan Science and Technology Company (Tokyo). X-ray tube voltage is 50 kV, tube current is 50 mA, and diaphragm aperture is 20 mm.

The catalyst phase structure was determined by D/max-3C X-ray diffractometer (XRD) produced by Japan Science Corporation (Tokyo) with the following settings: Cu target, Kα radiation, tube voltage 40 kV, tube current 20 mA, scanning range: 5°–50°, and scanning speed 4°/min.

The specific surface area and pore volume of the catalyst were measured by Micromeritics ASAP 3000 automatic physical adsorption instrument produced by Micromeritics Instruments Corporation (Norcross, GA, USA). The surface area was deduced from the adsorption isotherms using the BET equation. The samples were degassed at 300°C for 4 h (residual pressure value was 0.13 Pa), and then nitrogen physical adsorption was determined at -196°C.

The catalyst surface morphology was observed by ULTRA PLUS thermal field emission scanning electron microscopy produced by Zeiss optical instruments (Oberkochen, Germany). Secondary electron resolution was 1.0 nm (15 kV) 1.9 nm (1 kV); electron gun: LaB6 thermal field emission electron gun; acceleration voltage: 0.1–30 kV; magnification: ×12 ∼ X10,000.

### Catalyst Cracking Activity and Selectivity

Cracking performance of different catalysts was tested in an Advance Cracking Evaluation (ACE) unit developed by Kayser Technology Inc. Company, USA. Reaction conditions were the following: the amount of catalyst added was 9 g. The reaction and regeneration temperature were 530 °C and 715°C, respectively. Gaseous products were analyzed using a GC-3000 online chromatograph produced by INFICON Company (New York, USA) according to the UOP method 539. GC-3000 used four chromatographic modules for detection, and the work temperature was 0°C–50°C. The carrier gases used were helium, hydrogen, nitrogen, and argon. Simulated distillation of liquid products was carried out using a7890B chromatograph produced by Agilent Technologies Inc. (California, USA) according to the SH/T 0558 procedure. The working environmental temperature of Agilent 7890B was 15°C–35°C. Retention time repeatability was <0.0008 min. Carrier and makeup gas settings were selectable for helium, hydrogen, nitrogen, and argon/methane. Coke deposited on the catalyst was quantified with a CO_2_ analyzer produced by Servomex Group Co., Ltd (Sussex, England). The detection range of CO_2_ analyzer was 0–20%. When the detection value was lower than 0.4%, the regeneration step was considered to be completed. Conversion and yields of dry gas (H_2_ + C_1_ +C_2_), LPG (liquefied petroleum gas) (C_3_ +C_4_), gasoline (C_5_ < bp < 221°C), LCO (light cyclic oil) (221°C < bp < 343°C), HCO (heavy cyclic oil) (bp > 343°C), and coke were calculated.

Five different amounts of iron deposited catalysts were tested in the ACE unit. The adjustment of the catalyst to oil (C/O) ratio could be realized by changing the catalysts’ addition amounts under a fixed feed injection quantity. The conversion and product yield fitting curves were obtained on the basis of the evaluation data. The product selectivity of different iron deposited catalysts was compared under the same conversion.

## Results and Discussion

### Iron Deposition Rate on Catalyst

The amount of iron deposited on the catalyst was determined by XRF. Due to the existence of a certain amount of iron compounds in raw materials such as kaolin used in the preparation of the catalyst, the existing iron content in the catalyst should be deducted when calculating the iron deposition on the catalyst surface (Brandt et al., 2019). The results are shown in Table 2.

**TABLE 2:** Iron deposition of deactivated catalysts.

Sample	Fe on catalyst (μg/g)	Target increment (μg/g)	Actual increment^a^ (μg/g)	Deposition rate%
LE-0	1,881	0	53	-
LE-1	6,728	5,000	4,900	98.0
LE-2	9,228	7,500	7,400	98.7
LE-3	11,698	10,000	9,870	98.7
LE-4	13,518	13,000	11,690	89.9

^a^ Actual increment is equal to the amount of iron deposited on the sample minus that on the CAT-BASE.

LE-0 is obtained by cyclic deactivation of feed oil without iron spiking. Since the iron content in the feed oil is 0.62 μg/g, the amount of iron on the catalyst increased slightly to 53 μg/g after 200 cycles of cracking reaction. When the target amount of iron deposition is less than 10,000 μg/g, the iron deposition rate on the catalyst is more than or equal to 98%. When the target amount of iron deposition is 13,000 μg/g, the iron deposition rate on the catalyst decreased to less than 90%. Due to the high content of iron naphthenate in the feed oil (1,128 μg/g), some of the iron naphthenate may have been decomposed at the reaction temperature of 530°C before participating in the cracking reaction on the catalyst surface.

### Effect of Iron Deposition on Phase Structure of Catalyst

**FIGURE 1:**
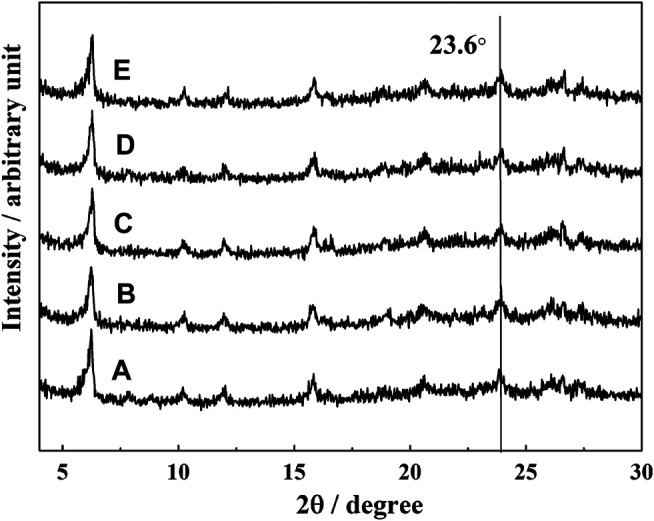
XRD patterns of catalysts with different iron content: **(A)** LE-0, **(B)** LE-1, **(C)** LE-2, **(D)** LE-3, and **(E)** LE-4.

The phase structure of the catalyst with different amounts of iron deposition was studied by XRD. The results are shown in Figure 1.

There were diffraction peaks at 2θ values of 6.2°,18.7°,20.3°, 23.6°, and 26.9°, which correspond to the characteristic peaks of (111), (331), (333), (533), and (642) of FAU zeolite (Lahnafi et al., 2020). With the increase of iron deposition on the catalyst, the position and peak intensity of each diffraction peak, especially the typical characteristic peak of Y zeolite at 2θ value of 23.6°, did not change significantly. This indicates that the iron deposited on the catalyst did not damage the crystal structure of Y zeolite.

### Surface Area and Pore Volume

In order to investigate the effect of different iron loading on the surface area and pore volume of the catalyst, the pore structure properties of the samples were analyzed by automatic physical adsorption instrument. The results are shown in Table 3.

**TABLE 3:** Surface area and pore volume of catalysts with different iron content.

Sample	BET surface area /(m^2^/g)	Zeolite surface area /(m^2^/g)	Matrix surface area /(m^2^/g)	Pore volume /(cm^3^/g)
LE-0	125.3	79.0	46.3	0.21
LE-1	123.8	79.0	44.8	0.21
LE-2	118.4	77.0	41.4	0.20
LE-3	108.3	73.7	34.6	0.17
LE-4	91.0	68.0	23.0	0.16

The surface area and pore volume decreased with the increase of iron deposition. When the iron deposition increased from LE-0 to LE-4, the surface area and pore volume decreased by 34.3 m^2^/g and 0.05 m^2^/g, respectively. When the iron deposition increased from LE-0 to LE-1, the surface area and pore volume of the catalyst were not affected significantly. The surface area decreased by only 1.5 m^2^/g, and the pore volume did not change. However, when the iron deposition was more than 7,400 μg/g, the surface area decreased by 27.4 m^2^/g, and the pore volume decreased significantly.

According to the surface area data of zeolite and matrix in Table 3, the matrix was more seriously affected by iron contamination. Compared with LE-0, the zeolite surface area retention of LE-4 was 86.1%, and the matrix surface area retention of LE-4 was 49.7%. This indicates that the pore closing effect of iron deposition on the catalyst is mainly reflected in the influence on the matrix.

Within the industrial FCC unit, the metals were deposited on the equilibrium catalyst usually with different deposition profiles. For example, nickel is known to deposit on the outer part of the catalyst (Meirer et al., 2015). Vanadium mainly diffuses from the outside to the inside (Cristiano-Torres et al., 2008; Pan et al., 2015). Iron does not migrate to the interior of the catalyst but exists in the depth of 1–5 μm (Yaluris et al., 2004; Wise et al., 2016; Ihli et al., 2017). Iron is mixed with silicon, calcium, sodium, and other elements to form a low melting point phase, which blocks the pore structure of the catalyst and forms the so-called iron nodules (Yaluris et al., 2004). The increase of iron nodules is the main reason for the decrease of surface area and pore volume of iron contaminated catalyst.

### Iron Contaminated Catalysts Morphology

**FIGURE 2:**
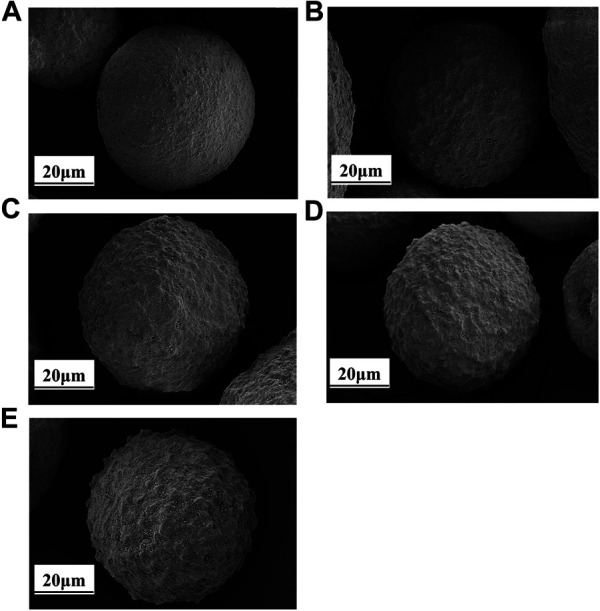
SEM of catalysts with different iron content: **(A)** LE-0; **(B)** LE-1; **(C)** LE-2; **(D)** LE-3; **(E)** LE-4.

The surface morphology of the catalysts with different iron deposition amounts was characterized by SEM, and the process of iron nodules on the catalyst surface with the increase of iron deposition was clearly observed. The details are shown in Figure 2.

Although 1,828 μg/g iron deposition can be detected on the catalyst LE-0, iron nodules were not observed on the surface of the catalyst because the iron was dispersed in kaolin during the preparation of the catalyst (Brandt et al., 2019).

More iron nodules can be observed with the increase of iron deposition on the catalyst. When the amount of iron deposition was 4,900 μg/g, a few nodules were formed on the surface of catalyst LE-1. The morphology of the catalyst was relatively complete. When the amount of iron deposition was 7,400 μg/g, the number of iron nodules on catalyst LE-2 increased more obvious. The surface area and pore volume of the catalyst LE-2 began to decrease to a certain extent due to the pore closing effect of iron nodules. When the amount of iron deposition reached 9,870 μg/g, the iron nodules on the surface of catalyst LE-3 were quite obvious and cover the whole catalyst.

When the amount of iron deposition reached 11,690 μg/g, the surface morphology of catalyst LE-4 was damaged most seriously by iron. Compared with the point distribution of iron nodules on the surface of catalyst LE-3, the nodules on catalyst LE-4 showed a block distribution. This may be due to the interweaving of high and low melting point phases on the catalyst surface, and the low melting point phases continued to collapse with the increase of iron deposition, resulting in the aggregation of high melting point phases in the adjacent region and then forming the distribution of iron nodules (Yaluris et al., 2004). The pore structure of the catalyst was seriously blocked under this amount of iron deposition. This conclusion can also be confirmed by the influence of different iron deposition on the surface area and pore volume of the catalyst, as shown in Table 3.

### Iron Effects on Catalyst Product Yields

**FIGURE 3:**
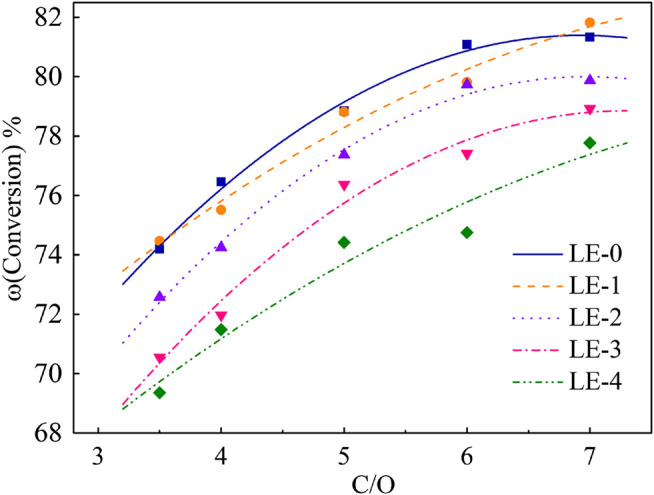
Conversion changes of catalysts with different iron content.

The performance of different iron deposition catalysts was evaluated by ACE unit. The conversion curves are shown in Figure 3.

C/O rate was chosen as 3.5, 4.0, 5.0, 6.0, and 7.0. It can be seen from Figure 3 that the increase of iron deposition leads to the decrease of conversion in the same range of C/O rate. The conversion of LE-1 decreased by 0.31% on average compared with LE-0, which indicated that the low iron deposition had little effect on the catalyst. The conversion of LE-4 decreased by 4.83% on average, which means that high iron deposition had the most significant contamination effect on the catalyst.

**FIGURE 4:**
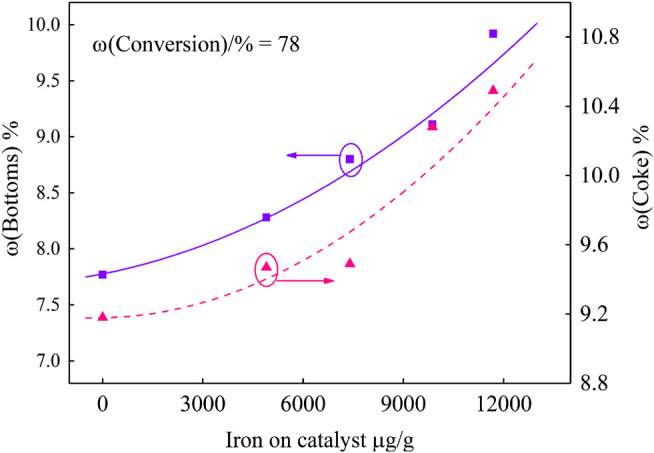
Bottoms and coke yields of catalysts with different iron content.

**FIGURE 5:**
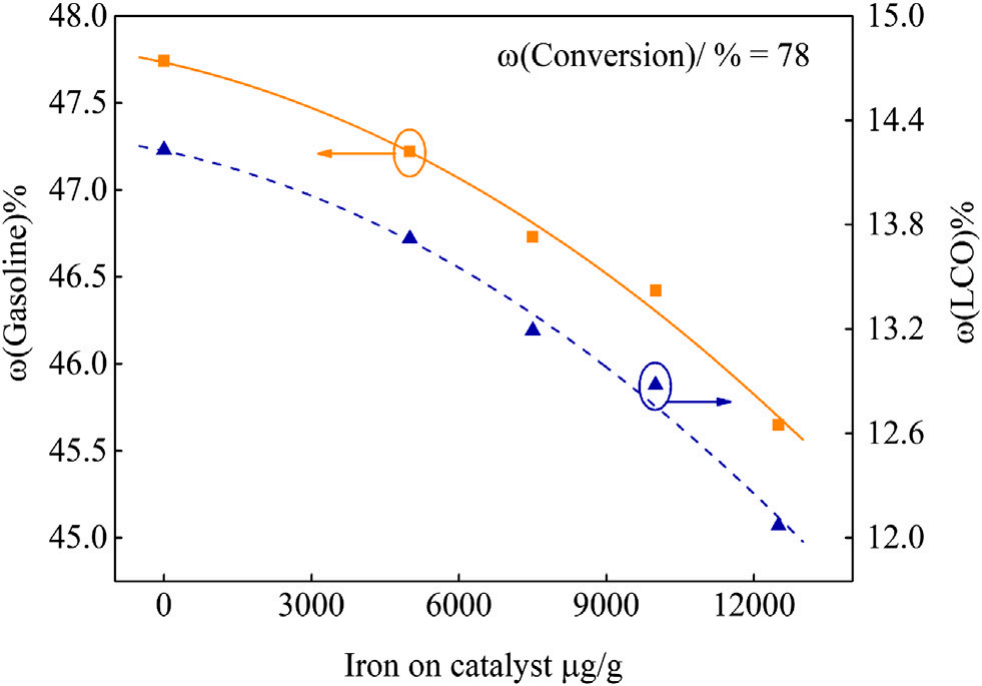
Gasoline and LCO yield of catalysts with different iron content.

In order to investigate the effect of different iron deposition on the catalyst performance, the product yield at fixed conversion was obtained by fitting curve. The results are shown in Figure 4 and Figure 5.

It can be seen from Figure 4 that, under the same 78 wt.% conversion, more and more iron deposits lead to higher yields of bottoms and coke. When the iron deposition increased from 53 μg/g to 11,690 μg/g, the bottoms yields increased by 2.15%. The iron nodules covered on the catalyst surface and the catalyst pores were partly blocked, which led to the decrease of the catalyst bottoms cracking capacity. Bottom molecules were difficult to be further cracked, which resulted in the increase of bottoms yield. According to the coke yield curve, coke increased by 1.31%. On the one hand, this is due to the iron deposition on the catalyst surface, resulting in dehydrogenation and condensation reactions. On the other hand, in the FCC unit, it is critical that, during the time the feed molecules spend in the riser, they diffuse inside the particle and the products diffuse out. Closing of pores by the iron nodules on the catalyst restricts the diffusion of feed molecules, which leads to excessive cracking reactions, and secondary reactions such as hydrogen transfer and condensation occur in the pore, which eventually results in the increase of coke yield (Meyers 2015; Chen and Xu, 2018).

It can be seen from Figure 5 that, under the same 78 wt.% conversion, more iron deposition leads to the decrease of gasoline and LCO yields, and their selectivity all becomes poor. When the iron deposition on the catalyst reached 11,690 μg/g, the gasoline and LCO yields decreased by 2.59% and 2.16%, respectively.

The special pore structure in the catalyst has a great influence on the yield of gasoline and LCO. When there are enough zeolite pores (about 0.75 nm) in the catalyst, and the pores are unobstructed and have good accessibility, gasoline selectivity will increase. When the number of secondary pores (>2 nm) in the catalyst is abundant, it can not only be used to crack larger molecules, but also provide pores for molecules to enter zeolite pores for re-cracking, so the LCO selectivity is increased (Chen and Xu, 2018). According to the analysis of the influence of iron deposition on the catalyst pore structure, with the increase of iron deposition, the catalyst surface is gradually covered by iron nodules. The pore structure which is beneficial to the formation of gasoline and LCO is destroyed, which results in the decrease of gasoline and LCO yields.

## Conclusion

The iron deposited on the catalyst did not damage the crystal structure of Y zeolite. The surface area and pore volume of the catalyst decreased with the increase of the amount of iron deposition. Part of the matrix pores was closed resulting in poor catalytic reaction performance. When the amount of iron deposited on the catalyst was more than 7,400 μg/g, iron nodules could be observed by SEM.

The increase of iron deposition on the catalyst will lead to the decrease of catalytic activity. When the iron deposition increased from 53 μg/g to 11,690 μg/g, the average conversion decreased by 4.83%. Under the same 78 wt.% conversion, the yields of bottoms and coke increased by 2.15% and 1.31%, respectively; the yields of gasoline and LCO decreased by 2.59% and 2.16%, respectively.

The iron nodules on the catalyst surface were simulated by using the cyclic deactivation method with multiple reaction and regeneration steps, which can reflect the real state of industrial iron contaminated equilibrium catalyst. It provides theoretical basis and experimental reference for further study of reaction mechanism. The product distribution evaluation based on cyclic deactivated catalyst is more accurate.

In our next research work, the iron contamination of FCC catalysts with different Si/Al ratios or different pore structures will be studied. With the help of cyclic deactivation method, we can understand the influence of iron on different catalysts more clearly.

## Data Availability

The original contributions presented in the study are included in the article/Supplementary Material. Further inquiries can be directed to the corresponding author.
